# Receptive Field Space for Point Cloud Analysis

**DOI:** 10.3390/s24134274

**Published:** 2024-07-01

**Authors:** Zhongbin Jiang, Hai Tao, Ye Liu

**Affiliations:** School of Automation and Artificial Intelligence, Nanjing University of Posts and Telecommunications, Nanjing 210023, China; 1322059108@njupt.edu.cn (Z.J.); 1021051806@njupt.edu.cn (H.T.)

**Keywords:** point cloud, receptive field, attention

## Abstract

Similar to convolutional neural networks for image processing, existing analysis methods for 3D point clouds often require the designation of a local neighborhood to describe the local features of the point cloud. This local neighborhood is typically manually specified, which makes it impossible for the network to dynamically adjust the receptive field’s range. If the range is too large, it tends to overlook local details, and if it is too small, it cannot establish global dependencies. To address this issue, we introduce in this paper a new concept: receptive field space (RFS). With a minor computational cost, we extract features from multiple consecutive receptive field ranges to form this new receptive field space. On this basis, we further propose a receptive field space attention mechanism, enabling the network to adaptively select the most effective receptive field range from RFS, thus equipping the network with the ability to adjust granularity adaptively. Our approach achieved state-of-the-art performance in both point cloud classification, with an overall accuracy (OA) of 94.2%, and part segmentation, achieving an mIoU of 86.0%, demonstrating the effectiveness of our method.

## 1. Introduction

A point cloud is a simplified representation of an object in three-dimensional space. In recent years, with the development of technologies such as autonomous driving [1,2], 3D modeling [3], and remote sensing detection [4], point cloud analysis has become a hot topic in the field of 3D vision. It has garnered widespread attention from both the scientific and industrial communities [5,6,7,8].

The point cloud is composed of an unordered and irregular set of points $¶\in R^{N\times 3}$, exhibiting rotation and permutation invariance, collectively referred to as irregularity. Unlike 2D image data arranged on a pixel grid, 3D point cloud information is a continuous collection embedded in space. This makes it nearly infeasible to directly apply well-established classification and segmentation models from the image processing domain.

In this paper, we primarily focus on two typical tasks in point cloud processing: point cloud classification and segmentation. Traditional methods for solving these problems have relied on manually crafted features to capture the geometric attributes of point clouds [9,10]. Since 2017, the success of deep neural networks in image processing has sparked interest in feature learning methods for point cloud data based on various neural network architectures. Deep point cloud processing and analysis methods are rapidly advancing, surpassing traditional approaches in various tasks [11].

Due to the irregularity of point cloud data, adapting deep learning frameworks for point cloud processing is challenging. Before the emergence of PointNet [12], there were two mainstream approaches. One method involves projecting point cloud data onto different planes, and then, applying deep learning methods designed for 2D images [13,14,15,16]. The drawback of this approach is that projection can lead to occlusion and information folding, resulting in the loss of local features within the point cloud. The other method is voxel-based [17,18,19], which converts the original point cloud into 3D grid data, where each point corresponds to a voxel (a small cube) in the grid. Subsequent processing involves using 3D convolutions. This method’s effectiveness is highly dependent on the choice of voxel size and incurs significant computational and memory overheads, making it challenging to capture detailed features.

The success of deep neural networks in image processing has spurred the development of various neural network-based methods for learning features from point cloud data. Methods for deep point cloud processing and analysis are rapidly evolving, surpassing traditional approaches in various tasks. Currently, the most mainstream deep neural networks suitable for point clouds almost all originate from PointNet [12]. PointNet designed a unique feature extractor that processes individual points at a local scale, maintaining the inherent irregularity of point clouds and directly operating on the raw point cloud data. Subsequent models such as PointNet++ [20], DGCNN [21], PointConv [22], and RSCNN [23] have largely continued PointNet’s design approach. They extract point cloud information through feature extractors, and then, process the aggregated feature information using different network architectures. However, when dealing with unstructured data like point clouds, traditional convolutional neural networks often face challenges due to the irregularity and invariance in point cloud data representation. Point cloud data comprise an unordered set of points, lacking a well-defined structure like images, making traditional convolution operations difficult to apply directly. Furthermore, transformations such as rotation, translation, and scaling of point cloud data should not alter their meaning, posing additional challenges for traditional convolution operations in meeting the invariance requirements of such data.

To address these issues, researchers have begun exploring deep-learning-based methods for point cloud analysis. These methods attempt to mimic convolution operations in traditional image analysis to achieve similar feature extraction and pattern learning on point cloud data. Representative methods include set abstraction in PointNet++ [20], edge convolution in DGCNN [21], X-Conv in PointCNN [24], etc.

These methods typically simulate the size of convolution kernels by defining local neighborhoods, similar to convolution operations in CNNs. These local neighborhoods determine the range observed by the convolution operation, i.e., the receptive field. In point cloud data, the size of the receptive field directly affects the model’s understanding and description of point cloud structures. Larger neighborhood ranges allow the model to capture broader contextual information, thereby achieving a more comprehensive grasp of global morphology and relationships. On the other hand, smaller neighborhood ranges are more conducive to detailed local feature extraction, enabling the model to identify and describe fine structures and patterns in point clouds more precisely. However, current methods often manually set the neighborhood size to define the receptive field range, such as setting the radius of set abstraction in PointNet++ [12] or the value of k in DGCNN [21].

To enable the network to autonomously learn and determine the range of the receptive field, we introduce a new concept called receptive field space. Receptive field space no longer views the receptive field as a single size and shape but considers it as a continuous range covering multiple scales and levels of local and global information. In receptive field space, receptive fields of different sizes and shapes are regarded as dimensions in feature space. By establishing the range of receptive fields in this dimension, the network can simultaneously consider and utilize feature information at different scales and levels. Thus, the neural network can comprehensively understand and represent the structure and relationships of the input data.

In the construction of receptive field space, we employ a simple yet efficient computational method to ensure that computational complexity does not exponentially increase with an increase in receptive field sampling density. This computational method fully utilizes the locality and sparsity of the data, effectively capturing feature information at different scales and levels while maintaining computational efficiency.

After constructing the receptive field space, we further propose a new receptive field attention mechanism called spatial-receptive field co-attention (SRCA), aiming to enable the network to adaptively focus on important receptive field ranges. The core idea of this mechanism is to dynamically adjust the receptive field range in feature space by introducing attention mechanisms, thereby achieving a more effective representation and processing of input data. The operation of the receptive field attention mechanism is similar to attention mechanisms: it allows the network to automatically adjust and allocate receptive field ranges of different sizes based on the features of the input data to focus on important areas. Specifically, by introducing attention mechanisms at different levels of the network, the importance weights of each position or feature channel can be calculated based on the current task and features of the input data. These weights can then be used to aggregate feature representations within the receptive field and generate the final output. This mechanism endows the network with the ability to autonomously adjust granularity, thereby better adapting to the feature extraction and pattern learning requirements at different scales.

This research method has been evaluated on two tasks: point cloud classification and part segmentation. The experimental results demonstrate that the method achieves state-of-the-art performance on both tasks and exhibits higher accuracy and faster inference speed compared to other methods with similar performance. Additionally, the method has relatively fewer parameters, making it more feasible and efficient for practical applications.

Our main contributions are:

- We introduce the concept of receptive field space and propose a simple and efficient method for constructing receptive field space. The introduction of receptive field space enables neural networks to learn and adjust receptive field ranges more flexibly, allowing them to adapt to features at different scales and levels.
- We propose a receptive field attention mechanism, which endows neural networks with the ability to autonomously learn and determine the receptive field range. This mechanism enables the network to dynamically adjust and focus on important receptive field ranges based on the features of the input data and task requirements, thereby improving the performance and generalization ability of the network.
- We extensively analyze and test this method, achieving state-of-the-art performance in point cloud classification and part segmentation tasks.

## 2. Related Works

**Hand-crafted features for point clouds:** Point clouds possess two main characteristics, rotation invariance and permutation invariance, collectively referred to as the unordered nature of point clouds. There is no inherent order between the arrangement of points, and swapping the order of points has no effect. Various tasks in point cloud processing and analysis, including segmentation, classification, matching, completion, and more, require constructing local features to describe the features of the point cloud. Numerous papers in computer vision and graphics have proposed local feature descriptors suitable for different problems and data structures in point clouds.

Broadly speaking, point cloud processing can be divided into intrinsic and extrinsic descriptors.

Intrinsic descriptors refer to the local characteristics of point cloud data, typically represented through relationships or statistical properties between points. For instance, the 3D shape context [25] is used to describe the local shape features of each point in a point cloud. It computes a shape context histogram for each point based on the distances and orientation relationships between point pairs in local point cloud information. Spin images [26] are used to describe the local surface shape of each point in a point cloud. They project point cloud data onto a fixed orientation grid and compute surface statistics for each direction.

Extrinsic descriptors pertain to the overall geometric and spatial properties of the point cloud data in three-dimensional space. For example, in 3D object recognition [27], they describe boundaries or boundary points in the overall point cloud to differentiate between different objects or shapes. Point feature histograms [10] describe the geometric relationships between point pairs in the overall point cloud by computing histograms of geometric features for each point pair, including normals, curvature, and relative positions between points.

These traditional methods have largely inspired numerous deep learning approaches tailored for point clouds.

**Deep learning on point clouds:** Methods for processing 3D point clouds using learning-based approaches can be categorized into the following types: projection-based, voxel-based, and point-based networks.

For handling irregular inputs like point clouds, an intuitive approach is to transform the irregular representation into a regular one. Given the widespread use of CNNs in 2D image processing, some methods [28,29,30,31,32] employ multi-view projection. In these methods, 3D point clouds are projected onto various image planes, and then, 2D CNNs extract feature representations from these image planes. Finally, multi-view feature fusion is performed to form the final output representation. However, in these methods, the geometric information within the point cloud is collapsed during the projection phase, and the sparsity of the point cloud is not well utilized. Moreover, the choice of projection planes can significantly affect recognition performance, and occlusions in three dimensions may hinder accuracy.

Another approach to processing point cloud data is 3D voxelization followed by 3D convolution using transformer-based methods [17,18,33]. However, as the number of voxels increases, the corresponding resolution grows exponentially, leading to significant computational and memory overheads. Moreover, since the point cloud is quantized into a voxel grid through certain methods, the inherent geometric details of the point cloud can be lost, resulting in a loss of accuracy. The effectiveness of voxel-based methods highly depends on the choice of voxel size. Improper voxel size selection can lead to information loss or over-sampling.

Unlike projecting or quantizing irregular point clouds onto 2D or 3D regular grids, researchers have designed deep network architectures that directly take point clouds as sets embedded in continuous space. PointNet [12] uses permutation-invariant operators like point-wise MLPs and pooling layers to aggregate features within the set. PointNet++ [20] applies these ideas within a hierarchical spatial structure to enhance sensitivity to local geometric layouts. DGCNN [21] connects the point set into a graph and performs message passing on this graph. It executes graph convolution on a KNN [34] graph, using EdgeConv to construct a local graph that generates edge features describing the relationships between points and their neighbors. Point Transformer [35] introduces the vision transformer structure suitable for 2D images, utilizing self-attention mechanisms to learn complex relationships and dependencies between points in point cloud data. This effectively integrates global and local information within point cloud data, improving feature extraction and representation learning performance. PointMlp [36] constructs an efficient pure MLP deep connectivity structure, performing feature extraction, mapping, and transformation through multiple fully connected layers.

Therefore, we believe that due to the unique complexity of point clouds, carefully designing feature extractors for local geometric structures can significantly improve the accuracy of tasks such as classification and segmentation of point clouds.

**Spatial attention and channel attention:** Spatial attention primarily focuses on different spatial locations within feature maps, adaptively emphasizing and adjusting the importance of this positional information. The core idea of this attention mechanism is to capture key information and important structures in images by learning the weights of each spatial location, thereby optimizing the generalization ability of the model.

On the other hand, channel attention concentrates on different channels within the feature map. It learns the weights of each channel to adjust the importance between different channels. Like spatial attention, it can also adjust the weights of individual channels through convolutional neural networks to extract key features from point clouds, improving the performance of the model in tasks related to detecting and segmenting spatially related positions.

Combining them results in the convolutional block attention module [37]. This model first applies spatial attention to adjust the importance of different spatial locations in the feature maps. Then, it applies channel attention to adjust the importance of different channels within the feature map. Finally, the feature maps adjusted by both attention modules are combined to obtain the final feature representation.

## 3. Method

**Figure 1:**
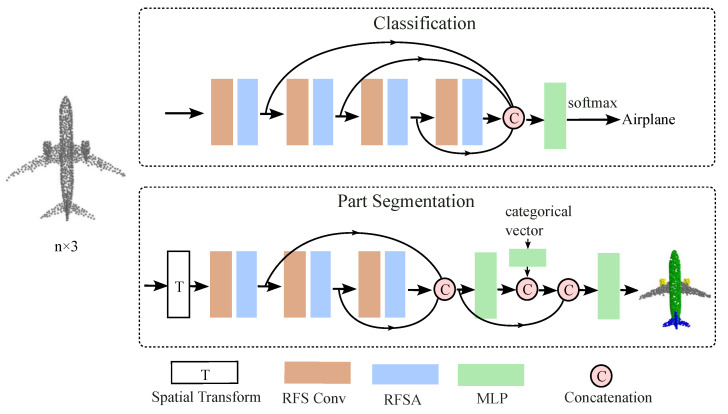
Overall diagram of our proposed method for two tasks: classification and shape part segmentation. RFS Conv stands for receptive field space convolution, RFSA stands for receptive field space attention, MLP stands for multi-layer perceptron.

The overall structure of the proposed method for classification and shape part segmentation is shown in Figure 1. The network is composed of several repeated units, each unit including an RFS convolutional module and an RFS attention module.

### 3.1. Receptive Field Space Convolution

The receptive field (RF) refers to the specific region to which a neuron in a neural network is sensitive. In the context of convolutional neural networks (CNNs), the receptive field of a neuron denotes the area of the input image that influences the activation of that neuron. As data propagate through various layers of CNNs, the receptive field of neurons gradually increases, enabling them to capture more contextual information from the input.

In point cloud analysis, due to the non-structural and sparse nature of point cloud data, traditional convolution operations are no longer applicable. Therefore, most deep-learning-based point cloud analysis methods attempt to construct convolution operations similar to CNNs on 3D point clouds to effectively process and extract features from the data. In this process, the size and position of the receptive field define which part of the input the neuron “observes” or “perceives” to make decisions, which is crucial for understanding and pattern recognition in point cloud data.

To further enhance the processing capability of point cloud data, we introduce the concept of receptive field space. Receptive field space is formed by stacking feature maps generated by performing convolution operations on a series of different receptive field ranges.

Our definition of the stack of this series of feature maps is as follows:

(1) $$F^{\prime }=\left[\varphi_{r_{1}}\left(F\right),\varphi_{r_{2}}\left(F\right),\ldots ,\varphi_{r_{S}}\left(F\right)\right]$$

where φ is some kind of basic convolution operator, and $r_{1},r_{2},\ldots ,r_{s}$ is a set of increasing receptive field ranges. […] is the feature stacking operation. F is the input points’ embeddings of shape N×D, N is the number of points, and D is the dimension of the embedding. The output is N×D×S, with a new feature dimension: the receptive field dimension.

This construction method of receptive field space allows the network to simultaneously consider and utilize multi-scale feature information, thereby improving the ability to extract features and learn patterns from point cloud data.

EdgeConv [21] is a convolutional operation designed specifically for the characteristics of point cloud data, generating local features by utilizing the relationships between points. In this process, the concept of the receptive field plays a crucial role, determining the range that neurons can perceive when processing point cloud data. EdgeConv leverages the concept of the receptive field to enable neural networks to better understand the local structures and global relationships in point cloud data. Therefore, we adopt it as the basic convolution operator φ in our RFS convolution, which can be written as

(2) $$\varphi_{k}\left(F\right)=\max_{j\in N_{k}\left(i\right)}\left(\left[F_{i}-F_{j},F_{i}\right]\right)$$

**Figure 2:**
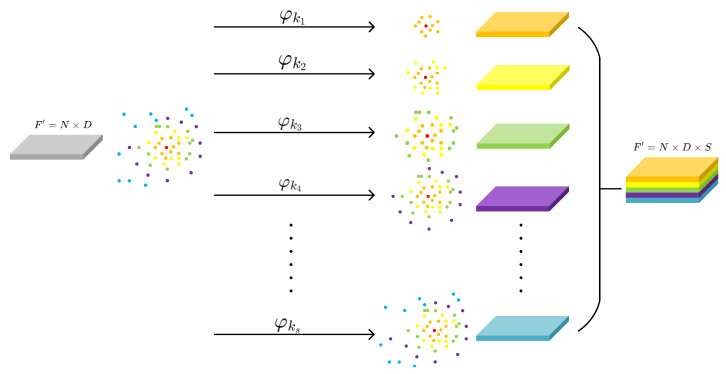
Aschematic diagram of RFS convolution.

Here, as shown in Figure 2, $N_{k}\left(i\right)$ represents the K-nearest neighbors of point *i*. For different choices of K, we can obtain different sizes of receptive field ranges. Therefore, we select an increasing sequence of K values $k_{1},k_{2},\ldots ,k_{s}$, constructing S RFS convolution operations with different receptive field sizes, each applied to F, resulting in F’.

### 3.2. Optimized Computation

RFS convolution is a novel convolution operation which involves performing the basic convolution operation φ on the input *S* times, which is quite time consuming. Thus, it may lead to a significant increase in computational complexity, affecting the computational efficiency of the network. Fortunately, we have discovered that there is a considerable amount of redundant computation involved in this process.

Firstly, for the K-nearest neighbors (KNN) operation, the following inclusion relationship exists:

(3) $$N_{k_{1}}\left(i\right)\subset N_{k_{2}}\left(i\right)\subset \ldots \subset N_{k_{S}}\left(i\right)$$

In other words, we only need to compute the maximum $N_{ks}\left(i\right)$ and retain the result, then the results of other neighborhoods can be obtained in O(1) time.

Next, we optimize the computation of the max operation within the neighborhood. If no optimization is performed, the time complexity of the max operation during the construction of the receptive field space is $O(k_{1}+k_{2}+,\ldots ,+k_{S})$.

We denote

(4) $$\max_{j\in N_{k_{1}}\left(i\right)}\left(\left[F_{i}-F_{j},F_{i}\right]\right),\max_{j\in N_{k_{2}}\left(i\right)}\left(\left[F_{i}-F_{j},F_{i}\right]\right),\ldots ,\max_{j\in N_{k_{S}}\left(i\right)}\left(\left[F_{i}-F_{j},F_{i}\right]\right)$$

as

(5) $$M_{1},M_{2},\ldots ,M_{s}$$

We partition the neighborhood into the following disjoint subsets:

(6) $$N_{k_{S}}\left(i\right)=N_{k_{1}}\left(i\right)\cap N_{k_{2}}\left(i\right)-N_{k_{1}}\left(i\right)\cap \ldots \cap N_{k_{S}}\left(i\right)-N_{k_{S-1}}\left(i\right)$$

Within each subset, perform the max operation to obtain a series of maximum values $m_{1},m_{2},\ldots ,m_{s}$; the time complexity here is $O(k_{s})$. Then, the calculations $M_{1}=m_{1}$, $M_{2}=\max\left(M_{1},m_{1}\right),\ldots ,M_{S}=\max\left(M_{S-1},m_{S}\right)$ can be carried out successively. The time complexity here is O(S), and the overall time complexity is reduced to $O(k_{s}+S)$.

By optimizing the computation process of constructing the receptive field space, specifically the RFS convolution process, we can improve the network’s computational efficiency without compromising the accuracy of the receptive field calculation.

### 3.3. Attention for Receptive Field Space

In the preceding sections, we introduced the RFS convolution operation and the optimized receptive field calculation method for point cloud data processing. Next, we will delve into the design and application of the receptive field attention mechanism.

By computing, we have constructed the receptive field space. We then design a receptive-wise attention mechanism to operate on this new feature dimension, allowing the network to adaptively focus on important receptive field ranges, thereby achieving self-adjustment of feature extraction granularity.

**Figure 3:**
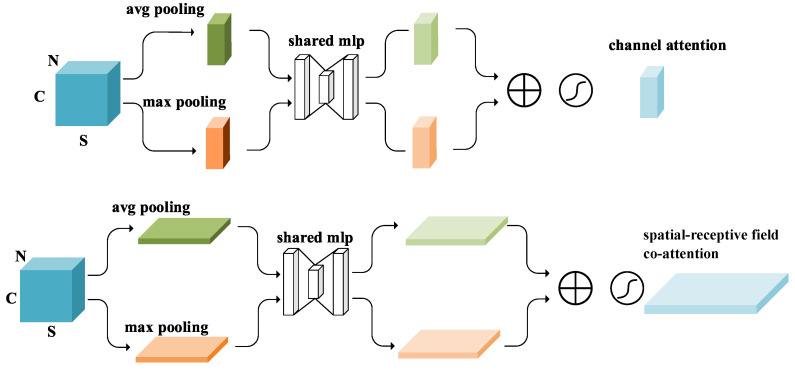
A schematic diagram of channel-wise attention and spatial-receptive field co-attention.

Here, we construct two types of attention: (1) channel-wise attention (CA) and (2) spatial-receptive field co-attention (SRCA). The schematic diagram of the two attentions is shown in Figure 3.

First, let us examine channel-wise attention (CA). Channel-wise attention primarily focuses on the relationships between different channels in the feature map. In our design, we first utilize average pooling and max pooling operations to compress the spatial and receptive field joint dimensions of the feature map, reducing redundant information and computational load. Then, we use a multi-layer perceptron (MLP) to integrate the compressed features and generate the channel attention weights.

Spatial-receptive field co-attention (SRCA) goes a step further by considering both spatial information and the receptive field range. In this design, we address not only the relationships between the channels in the feature map but also their relationships in the spatial and receptive field dimensions. Therefore, we combine spatial and receptive field information to design a co-attention mechanism. Specifically, we also use average pooling and max pooling to compress the channel dimension of the feature map, and then, utilize an MLP to generate the co-attention weights.

In constructing the overall attention mechanism, we drew inspiration from the CBAM (convolutional block attention module). The traditional spatial attention module CBAM was initially applied in image processing, and thus, includes two spatial dimensions. Following the CBAM approach, we implemented this traditional spatial attention module in point cloud processing, using CA+SA (channel attention and spatial attention) to achieve point cloud spatial attention. The feature maps used by these methods are derived from point cloud data processed through KNN (k-nearest neighbors). While effective, the performance was average.

Therefore, we changed our approach. We first extracted spatial features in layers within the receptive field space (RFS), and then, used CA and SRCA (spatial-receptive field co-attention) to process the data. This method allowed us to consider both spatial and receptive field dimensions simultaneously, thereby improving point cloud processing effectiveness.

RFS extracts spatial features in layers, enabling the model to better capture local and global features in the point cloud. After extracting features using RFS, we further applied CA and SRCA to enhance the feature representation. CA captures the importance of different channels in the point cloud, while SRCA provides a more precise attention mechanism by combining spatial dimensions and receptive field features.

Since images have two spatial dimensions, while our feature maps have one spatial dimension and one receptive field dimension, in form, our CA and SRCA are consistent with the channel-wise attention and spatial attention in CBAM. The specific expression is as follows:

(7) $$CA=\psi_{CA}\left(A\nu p_{sr}\left(F^{\prime }\right)\right)+\psi_{CA}\left(Map_{sr}\left(F^{\prime }\right)\right)$$

(8) $$SRCA=\psi_{SRCA}\left(Avp_{ch}\left(F^{\prime }\right)\right)+\psi_{SRCA}\left(Map_{ch}\left(F^{\prime }\right)\right)$$

where $Avp_{ch}$ and $Map_{ch}$ represent average and max pooling along the feature channel dimension, respectively. $\psi_{SRCA}$ denotes the MLP for SRCA attention. $Avp_{sr}$ and $Map_{sr}$ are average and max pooling performed jointly along the spatial and receptive field dimensions, respectively. $\psi_{CA}$ represents the MLP for CA.

Although formally these two attentions resemble CBAM, their content is indeed different. In CBAM, images have two spatial dimensions, while point clouds only have one spatial dimension, and we constructed a new receptive field dimension in point clouds. This enables our attention mechanism not only to focus the network on important feature channels and spatial positions but also to pay attention to significant receptive field ranges, thereby achieving adaptive adjustment of feature extraction granularity.

## 4. Experiments

### 4.1. Classification

We evaluate our classification model on the ModelNet40 [18] dataset, which comprises 12,311 CAD models meshed from 40 categories, including 9843 training samples and 2468 test samples. Each point cloud instance is uniformly sampled with 1024 points, utilizing only the spatial coordinates (x, y, z) for classification. Prior to training, we augment the point cloud data through random rotation and scaling.

The network structure used for the classification task is depicted in the upper part of Figure 1. In classification tasks, SRCA aggregates local features into global features through pooling operations for the classification of the entire point cloud. In segmentation tasks, SRCA not only utilizes local features but also propagates global features back to each point, allowing each point to have both local information and global context for refined segmentation. We employ a combination of four RFS convolution along with receptive-wise attention to adaptively extract and enhance the required spatial feature information across scale space. These four layers share a common fully connected layer and independently compute spatial feature information. For all RFS convolutions involved in the classification task, the value of k in the KNN is set to 5, 10, 15, and 20. After concatenating the four sets of high-dimensional spatial feature information, they are fed into a shared fully connected layer with 1024 dimensions. Subsequently, maxpool and avgpool are used to obtain all global features of the current point cloud information. Multiple MLPs are then used to transform these global features, followed by sigmoid normalization. We retrain the model on the entire training dataset and evaluate it on the test dataset.

We use SGD with a learning rate of 0.1 and employ cosine annealing [38] to reduce the learning rate to 0.001. The batch size is set to 16 with a momentum of 0.9.

Following the mainstream evaluation criteria for this dataset, we primarily focus on overall accuracy (OA) to compare our results with those of other models. We also compared the model’s inference speed and parameter volume. The OA (overall accuracy) metric is commonly used to evaluate the overall performance of a model. Parameter count reflects the complexity of the model, where more parameters indicate higher computational resource consumption and increased risk of overfitting. Speed typically reflects the efficiency of the model during the inference phase, with faster inference speed being crucial for real-time applications or tasks requiring large-scale data processing. The models we compare against include PointNet [12], PointNet++ [20], PointCNN [24], DGCNN [21], Point Transformer [35], CurveNet [39], PointNeXt [40], and PointMLP [36], We conducted tests using these open-source models.

The comparison results are shown in Table 1. Our model achieved excellent results on the classification task of this dataset, with an overall accuracy (OA) reaching 94.2%, comparable to the state of the art. Our model also performs well in terms of parameters and speed. Compared to PointMLP, our model outperforms it in both parameters and speed.

### 4.2. More Experiments on ModelNet40

#### 4.2.1. Ablation Experiments

This experiment aims to evaluate the impact of our method on the classification performance of the model. By gradually adding, removing, or altering certain layers in the network, we explore their influence on the overall performance of the model and verify their effectiveness in improving the key indicator OA (overall accuracy) in the classification task.

We conducted the experiment from two perspectives. In the first perspective, we modified the receptive-wise attention mechanism (receptive-wise attention) in Figure 2, replacing CA and SRCA with pure MLP structures, self-attention structures, and the CBAM we designed for point clouds. The first two structures are commonly used in mainstream point cloud segmentation methods, such as Point Transformer, CurveNet, and PointMLP, to enhance the extracted point cloud feature information. The latter method, CBAM, is widely used in image processing, and we designed a point cloud version to validate the effectiveness of SRCA.

In the second aspect of the experiment, we intended to test the impact of different depths of receptive-wise attention on the overall results, so we did not use all of receptive-wise attention to enhance feature information. We experimented by retaining n receptive-wise attention layers in order from deep to shallow. For example, using three receptive-wise attention layers means retaining the last three receptive-wise attention layers in the network and discarding the shallowest one. For experimental rigor, the RFS convolution preceding receptive-wise attention remains unchanged in both experiments. In all experiments, the information outputted by RFS convolution is set to the same adaptive multi-scale spatial features.

The experimental results are shown in Table 2. From the perspective of enhancing feature information, our receptive-wise attention mechanism, which uses CA+SRCA, significantly outperforms other point cloud classification methods such as multi-layer MLP and direct self-attention, as well as the CBAM we designed for point clouds. In terms of the effectiveness of dynamic adjustment at different levels, our receptive-wise attention mechanism is evidently suitable for all levels in the network; otherwise, the classification performance would decline.

#### 4.2.2. Feature Points

Feature points are those particularly highlighted by receptive fields of varying sizes within the receptive field space. In point cloud classification tasks, these feature points play a crucial role, representing the local features of the point cloud data, which include geometric information and the local structure of the object surface. RFS convolution extracts feature points based on different k values. By accurately extracting and describing these feature points, we can transform point cloud data into distinctive feature representations, providing essential input for classification models. The selection and description of feature points directly affect the model’s performance and accuracy, helping the model accurately recognize and classify different objects or scenes, thereby achieving more precise point cloud classification tasks.

**Figure 4:**
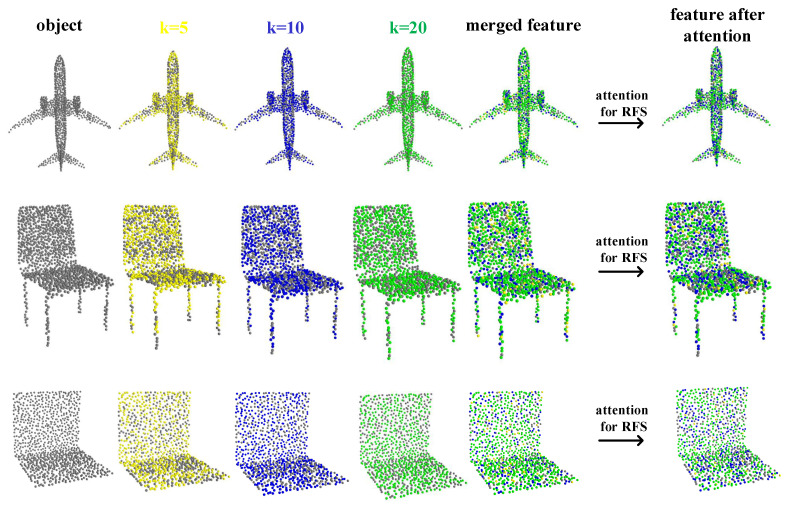
Visualization of feature point extraction and processing.

The introduction of receptive field space brings a new perspective to point cloud classification tasks. By constructing a series of receptive fields of different sizes to form a new feature dimension, the receptive field space allows the model to observe point cloud data comprehensively, thereby better understanding the data’s structure and features, as shown in Figure 4. This image illustrates the results of spatial feature point extraction by the receptive field space (RFS) and attention for receptive field space. The leftmost part shows the input point cloud data, followed by the spatial feature points extracted by the model under different receptive field sizes. These pieces of information are merged, and then, the attention for receptive field space adaptively adjusts the weights of the receptive field features, resulting in the output of adjusted spatial feature points. Clearly, our receptive field space can cover nearly the entire model and extract spatial feature information, which is further refined by channel-wise attention and spatial-receptive field co-attention for adaptive adjustment.

Simultaneously, the receptive field attention mechanism further optimizes the feature point extraction process. By allowing the model to adaptively focus on receptive field ranges with significant information, the receptive field attention mechanism enhances the model’s ability to capture and identify key points. This mechanism enables the model to focus more on crucial local feature points in classification tasks, thereby further improving the performance and accuracy of the classification model.

#### 4.2.3. Model’s Versatility

For point cloud classification tasks, the versatility of a model implies its ability to adapt to point cloud data with different quantities and densities, yielding satisfactory classification results across various scenarios.

**Figure 5:**
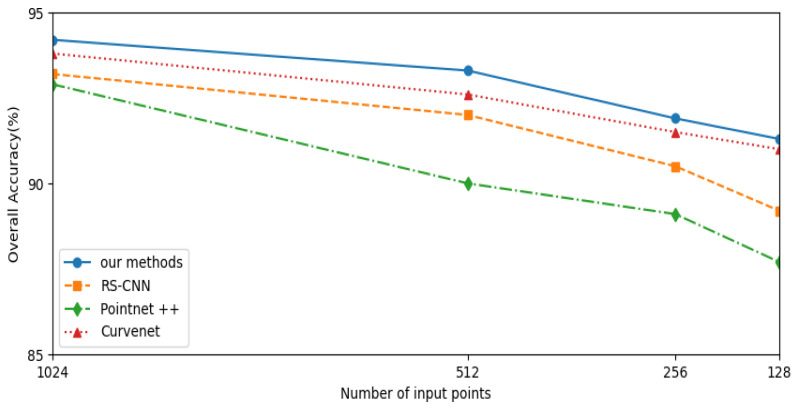
Comparison on sparser training and testing input point cloud.

Based on this concept, we conducted a series of experiments to validate the versatility of our point cloud classification model. This experimental design fully considered the diversity of point cloud data in real-world scenarios and evaluated the model’s performance from different perspectives. As shown in Figure 5, we trained the model using different numbers of input points (1024, 512, 256, and 128 points) and observed its performance in point cloud classification tasks under these varying input conditions. Regardless of whether the point cloud density was high or low, our model consistently achieved good classification results, confirming its excellent versatility.

#### 4.2.4. Receptive Field Space Visualization

Our model uses channel-wise attention and spatial-receptive field co-attention to process the features extracted by RFS convolution. This results in varying receptive field intensity representations for each point, highlighting the importance of different receptive field ranges and enabling the model to adaptively focus on key features at different scales.

**Figure 6:**
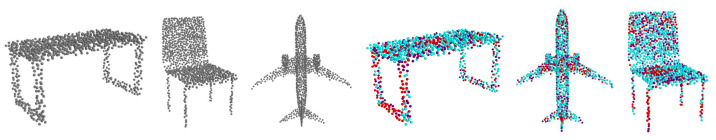
Each point in the point cloud is represented with different receptive field information.

As shown in Figure 6, our model achieves adaptive receptive field adjustment by representing the intensity of receptive field information for each point in high-dimensional space. In smoother regions, the receptive field is relatively larger because these areas require more contextual information to accurately describe their overall shape. In contrast, in regions rich in details, the receptive field is relatively smaller to capture local structures and fine variations more precisely. Consequently, our model can comprehensively understand the local structures and features at different scales within point cloud data, providing more accurate and comprehensive feature representations for subsequent tasks.

### 4.3. Part Segmentation

We evaluate our segmentation model on the ShapeNetPart dataset, which contains 16,881 shapes from 16 categories, annotated with a total of 50 parts. Most object categories are labeled with two to five parts. We use the average intersection over union (IoU) across all instances as the evaluation metric. For each point cloud instance, it is uniformly sampled with 2048 points.

The network architecture is shown in the lower part of Figure 1. After passing through the spatial transformation network, we utilize a combination of three RFS convolutions and receptive-wise attention to extract spatial feature information. A shared fully connected layer (1024) aggregates information from the preceding layers. Next, we append label information that has been embedded. Finally, three shared fully connected layers (256, 256, 128) are used to transform point features.

We also use SGD with a learning rate of 0.1 and employ cosine annealing to reduce the learning rate to 0.001. The batch size is set to 32 with a momentum of 0.9.

The final segmentation results are shown in Table 3, where we compare our results with PointNet [12], PointNet++ [20], DGCNN [21], SPLATNet [41], SpiderCNN [42], PointNeXt [40], and PointMLP [36]. While our segmentation results are not the best, they surpass the 86.0% threshold and rank among the top performers.

The results in Table 4 show the segmentation outcomes of our model on all instances in the ShapeNetPart dataset. Our model achieves excellent segmentation results for categories that emphasize spatial features, such as chair, airplane, skateboard, and table.

### 4.4. Optimized Computation Experiment

We conducted an optimized computation experiment using an NVIDIA RTX 3090 GPU, primarily aimed at testing the speed performance of the model during training and inference. To evaluate the effectiveness of the optimized computation method, we kept the experimental setup consistent with the previous classification and segmentation experiments, and set the parameter S in the receptive field space (RFS) to 4, using four spatial features for stacking in the experiment.

For the evaluation of the classification task, we continued to use the ModelNet40 [18] dataset, a widely used benchmark dataset for 3D object classification. For the evaluation of the segmentation task, we still used the ShapeNetPart [43] dataset, commonly used for 3D object part segmentation. We tested the results of both tasks before and after applying the optimized computation method.

As shown in Table 5, our optimized computation method with S = 4 demonstrated a significant improvement compared to the non-optimized version. The optimized method not only showed a noticeable increase in training speed but also exhibited a substantial advantage in inference speed. Specifically, in the classification task, the optimized model showed significant improvements in computational efficiency. In the segmentation task, the optimized model also demonstrated considerable improvements in processing time.

### 4.5. Visualization of Segmentation Results

In this section, we will detail the model’s segmentation process and visualize its segmentation results.

**Figure 7:**
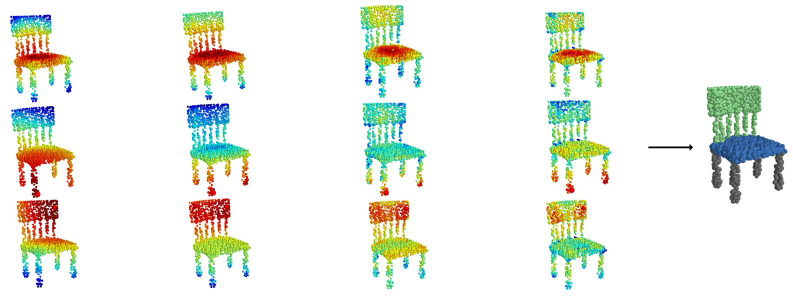
Visualization of segmentation process.

Figure 7 illustrates the visualization of the model’s segmentation process. Feature points from different parts are aggregated through multiple network layers to gather high-dimensional spatial feature information, ultimately focusing on specific parts. This process demonstrates the model’s refinement and optimization in the segmentation task.

**Figure 8:**
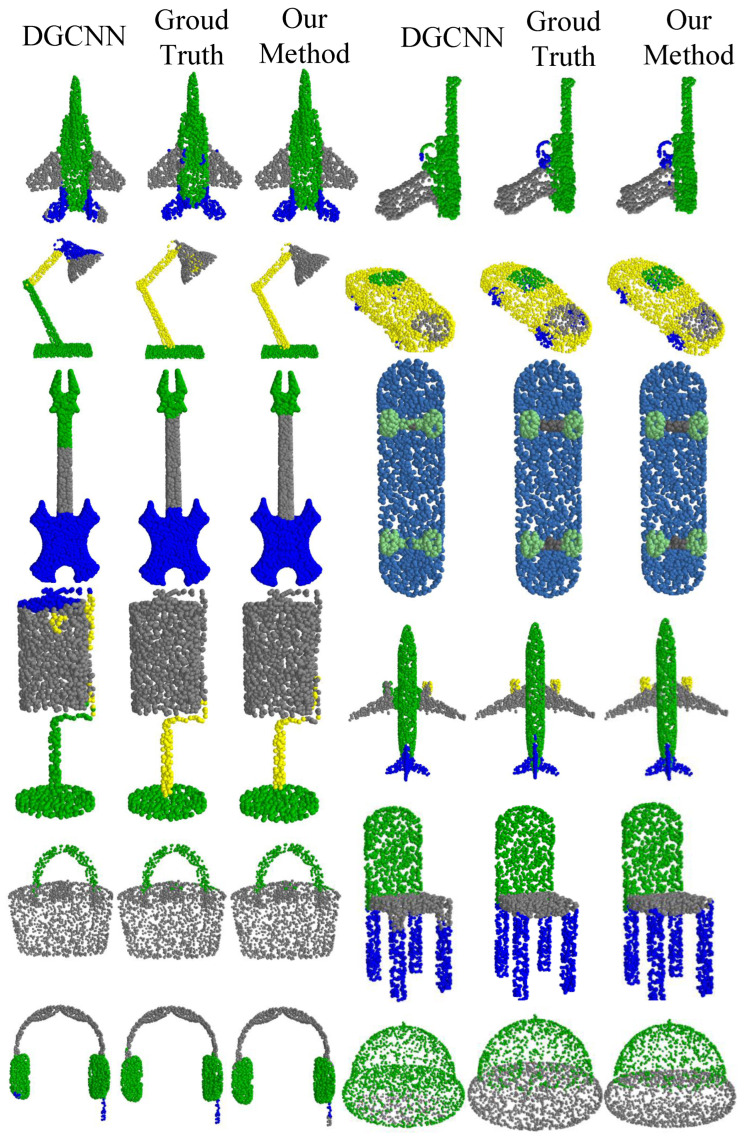
Visualization of segmentation results.

Figure 8 shows the final segmentation results. On the left are the segmentation results of DGCNN, a model that also utilizes spatial information. In the middle are the ground truth labels from the dataset, and on the right are the segmentation results obtained using our method. A clear comparison shows that our method outperforms others in terms of segmentation quality, exhibiting higher accuracy and reliability.

## 5. Discussion

In this work, we explored the receptive field space and proposed the receptive field attention mechanism based on it. We designed experiments to demonstrate the performance of this method in mainstream point cloud classification and segmentation tasks. Our experiments validated the feasibility of allowing the network to autonomously learn and determine the receptive field range. Our approach is not limited to traditional point cloud feature extraction methods but achieves more accurate and comprehensive capture of spatial local feature points by introducing receptive field space and the receptive field attention mechanism. This method enables the model to better understand the structure and features of point cloud data, thereby improving the effectiveness of classification and segmentation tasks.

By employing carefully designed feature extractors to extract and enhance features, we can more effectively accomplish classification and segmentation tasks. Through the introduction of receptive field space and the receptive field attention mechanism, we further optimize the feature extraction process, allowing the model to better focus on important receptive field ranges, thereby enhancing its ability to capture and identify key points.

However, we also recognize the challenges facing current point cloud classification and segmentation tasks. Firstly, the inherent noise and uncertainty in datasets make it difficult for models to learn and generalize accurately. Future improvements may require more complex representational capabilities to capture this diversity. Secondly, mainstream models have already identified optimal model structures and parameter settings for these datasets, and further improvements may require more domain-specific knowledge or innovative technologies. Finally, although our method can adaptively adjust the receptive field range to achieve autonomous granularity adjustment, the feature extraction method we use still belongs to traditional methods. We hope to innovate the extraction method in our future research.

## Figures and Tables

**Table 1:** Classification on the ModelNet40 dataset.

Method	OA (%)	Speed (ins./s)	Params (M)
PointNet [12]	89.2	2283	3.48
PointNet++ [20]	90.7	1268	1.48
PointCNN [24]	92.5	152	8.20
DGCNN [21]	92.9	535	1.80
PointTrans. [35]	93.7	90	2.94
CurveNet [39]	93.8	145	2.03
PointNeXt [40]	93.2	760	1.51
PointMLP [36]	94.2	120	12.36
Our method	94.2	476	1.82

**Table 2:** Ablation experiments: the “CBAM” was implemented by us in point cloud processing tasks based on our understanding of the CBAM method from image processing.

Number of Receptive-Wise Attention	MLP	CA+SRCA	Self-Attention	CBAM	OA (%)
4				✓	93.5
4			✓		93.9
4	✓				93.7
4		✓			94.2
3		✓			93.7
2		✓			93.3
1		✓			92.9

**Table 3:** Part segmentation results on the ShapeNetPart dataset.

Method	Inst.mIoU (%)
PointNet [12]	83.7
PointNet++ [20]	85.1
DGCNN [21]	85.2
SPLATNet [41]	85.4
SpiderCNN [42]	85.3
PointMLP [36]	86.1
PointNeXt [40]	86.3
Our method	86.0

**Table 4:** The segmentation results for all instances in the ShapeNetPart dataset. Red, blue, and green, respectively, represent the first, second, and third rankings of the data.

Method	Inst. mIoU	plane	bag	cap	car	chair	ear phone	guitar	knife	lamp	laptop	motor	mug	pistol	rocket	skate board	table
PointNet [12]	83.7	83.4	78.7	82.5	74.9	89.6	73.0	91.5	85.9	80.8	95.3	65.2	93.0	81.2	57.9	72.8	80.6
PointNet++ [20]	85.1	82.4	79.0	87.7	77.3	90.8	71.8	91.0	85.9	83.7	95.3	71.6	94.1	81.3	58.7	77.4	82.6
DGCNN [21]	85.2	84.0	83.4	86.7	77.8	90.6	74.7	91.2	87.5	82.8	95.7	66.3	94.9	81.1	63.5	74.5	82.6
SPLATNet [41]	85.4	83.2	84.3	89.1	80.3	90.7	75.5	92.1	87.1	82.6	96.1	75.6	95.2	83.8	64.0	75.5	81.8
SpiderCNN [42]	85.3	83.5	81.0	87.2	77.5	90.7	76.8	91.1	87.3	83.3	95.8	70.2	93.5	82.7	59.7	75.8	82.8
PointMLP [36]	86.1	83.5	83.4	87.5	80.5	90.3	78.2	92.2	88.1	83.9	96.2	75.2	95.8	85.4	64.6	83.3	84.3
PointNeXt [40]	86.3	83.9	83.6	86.2	81.1	90.5	77.3	91.5	88.4	82.3	95.9	77.5	95.6	84.4	66.1	83.5	84.4
Our Method	86.0	84.8	84.2	87.3	79.1	90.6	74.7	90.9	88.2	83.0	95.8	67.4	93.5	82.3	64.5	83.1	84.6

**Table 5:** Optimized computation experiment results.

	Optimized Computation Speed (ins./s)	Non-Optimized Computation Speed (ins./s)
**Classification Training**	185	67
**Classification Inference**	476	243
**Segmentation Training**	78	39
**Segmentation Inference**	245	134

## Data Availability

Data are contained within the article.
